# Supplementary material for: Anterior cingulate cortex causally supports flexible learning under motivationally challenging and cognitively demanding conditions
Source: PLoS Biol. 2022 Sep 6;20(9):e3001785. doi: 10.1371/journal.pbio.3001785 (PMC9481162; doi:10.1371/journal.pbio.3001785)
Supplement: S1 Text — Contains detailed methods for precise TUS neuronavigation, TUS simulations, and data analysis. (DOCX) [file pbio.3001785.s001.docx]

Supplementary Information for

The anterior cingulate cortex causally supports flexible learning under motivationally challenging and cognitively demanding conditions

Kianoush Banaie Boroujeni^1*^, Michelle K Sigona^3,4^, Robert Louie Treuting^4^, Thomas J. Manuel^3,4^, Charles F. Caskey^2,3,4^, Thilo Womelsdorf^1,4,*^

**Ethics Statement**.

All procedures were in accordance with the National Institutes of Health Guide for the Care and Use of Laboratory Animals, the Society for Neuroscience Guidelines and Policies, and approved by the Vanderbilt University Institutional Animal Care and Use Committee (M1700198-01).

**Neuro-navigation setup.**

An MR image was acquired before implantation of the head-post and a CT image was obtained containing the headpost with a fiducial array arm attached to it during the scan. The fiducial array in the CT scan was identical to its position during the neuro-navigation procedure in an experimental session. We co-registered MR and CT images into the same space (CT images were the fixed volume to prevent distortion of fiducial array space) using FSL [1]. 3D-Slicer software ([http://www.slicer.org](http://www.slicer.org/)), with Image-guided therapy (IGT) [2] extension modules were used for tracking and positioning the transducer. First, the co-registered brain images were imported to 3D-Slicer. Fiducial array points were marked on the images ordered from right to left. These points allowed reconstructing the relative distances of the sonication target locations in the brain from the fiducial array. At the beginning of each session, the fiducial array was mounted to monkey's headpost and fixed to the exact location as in the CT images. A stylus tool was then used to collect the physical fiducial array points in the same order as it was marked in the image space. After collecting the physical points, we transformed the physical space into image space. The focus to tracker transformation (which was used for tracking the focus of the transducer relative to the tracker attached to its enclosing cone) was also transformed with the same transformation to the image space [3,4]. We then navigated the focal point of the transducer (a circle with 3 mm radius) centered at the maximum focus estimated as sphere model (1-mm radius) placed at the focal maximum that was used for the location of targets in each area and hemisphere (these sphere models were held the same across all experiments for each monkey). Under the IGT module, we tracked the real-time distance of the center of the transducer focus from the target sphere model.

**Acoustic modeling of sonication beam**.

To validate sonication of the ACC and STR, we performed 3D numerical simulations of the acoustic propagation through the monkey skull using the open-source MATLAB toolbox k-Wave ([Http://www.k-wave.org](http://www.k-wave.org/)). A single-element transducer with a radius of curvature of 63.2 mm and an active diameter of 64 mm (H115MR, Sonic Concepts, Bothell, WA) and resonant frequency of 250 kHz was modeled as a spherical section. We used 3D-Slicer combined with optical tracking to position the transducer for simulations to recreate the optical tracking geometry. To localize the transducer relative to the transducer tracker, we manually aligned a transducer mask’s marked geometric focus created in k-Wave with the center of the focal location from reconstructed temperature images collected using a 2D gradient echo thermometry pulse. MR thermometry was performed on a 3.0T Philips Achieva 2D gradient echo thermometry pulse sequence with a multi-shot EPI readout factor of 3. Imaging parameters were 150 x 150 mm^2^ field of view (FOV), 112 x 112 matrix, 1.3 x 1.3 mm^2^ voxel size, 5 slices, 4 mm slice thickness, TE/TR 12/500 msec. A continuous wave sonication for 30 sec. at input voltage of approximately 1 MPa was used to generate several degrees of heating. This calibration was performed once to generate a transform with an accurate offset and rotation relative to the optical tracker. A CT image of the respective monkey was resampled using the Resample Image (BRAINS) module in Slicer so that the resolution and spacing matched the calibrated transducer mask or integration with simulations. The resampled data was exported to MATLAB to run acoustic simulations, using the CT to estimate the speed of the sound and density of the skull. The final spatial maps were imported back into 3D Slicer and overlaid for comparison with the estimated focus from the optical tracking and MR thermometry results. To account for heterogeneities of the monkey skull, a linear relationship between Hounsfield units (HU) from CT scans was used to calculate the speed of sound and density [5]. Brain tissue was assumed to have the same speed of sound and density as water. Values used included a padded grid size [Nx,Ny,Nz] of [384,288,280] with isometric voxels of 0.25 mm, attenuation = 8 dB/cm, power law absorption exponent of 1.1, $c_{min}=1480 m/s$, $c_{max}=3100 m/s$, $\rho_{water}=1000 kg/m^{3}$, and $\rho_{bone}=2100 kg/m^{3}$. Maximum pressure was recorded for every voxel in the simulation grid. The highest pressure was found at the skull, as it absorbs more sound than brain tissue. For each dataset, the spatial map cropped the maximum pressure of the skull so that an accurate root-mean-square (RMS) of the sound pressure could be calculated at the focus. The focal spot size of the sonicated area was calculated using $P>P_{max}/2$for half-maximum pressure (-3 dB; **S3C Fig**) and $P^{2}> P_{max}^{2}/2$ for half maximum intensity (-6 dB; **S3D Fig,** error bars showing standard error of the mean across sessions). The half-maximum pressure and intensity equations were used to create a mask of the focal spot volume and calculate the RMS pressure for each (**Fig 2A**).

From acoustic simulations we determined the spatial averaged pressure was 581.5 ± 95.8 kPa (NHP W: 623.38 ± 124.93 kPa, NHP I: 544.79 ± 31.16 kPa) at the striatum and 574.1 ± 56.0 kPa at the ACC (NHP W: 595.7 ± 46.3 kPa, NHP I: 555.3 ± 57.9 kPa). We used these pressures to estimate a mean attenuation of 51.9% for both NHPs (NHP W Striatum: 48.1%, NHP W ACC: 50.4%, NHP I Striatum: 54.6%, NHP I ACC: 53.7%). These values agree with prior estimations of macaque skull attenuation based on water bath measurements previously reported by our group and others [6,7].

To assure that the effect is not attributed to modulation in orbitofrontal cortex (OFC) which appears aligned with the TUS orientation in **Fig 2A** we ran a control analysis on ACC-TUS sessions. From acoustic simulations we evaluated the pressure at the orbitofrontal cortex and compared it with the pressure at the anterior cingulate cortex. We centered a cube with an edge length of 4 mm around labeled targets at the ACC and OFC and calculated the spatial average within the volume (**S3F Fig**). The mean spatial averaged pressure (mean ± standard deviation) for both NHPs was 574.1 ± 56.0 kPa at ACC (NHP W: 595.7 ± 46.3 kPa, NHP I: 555.3 ± 57.9 kPa) and 158.5 ± 56.6 kPa at OFC (NHP W: 137.3 ± 46.4 kPa, NHP I: 177.0 ± 59.1 kPa).

**Analysis of behavioral learning and fixational sampling.**

The improvement of accuracy over successive trials relative to the beginning of a learning block reflects the learning curve, which we computed with a forward-looking 12-trial averaging window in each block (**Fig 1E**). We defined learning speed in a block as the number of trials needed to reach criterion accuracy of ≥80% correct trials over the subsequent 12 trials (**Figs 2B, 2C**, **S1C**, **and S2C**). When monkeys did not reach the learning criterion at the end of a block, we estimated the trial number the monkeys would have learned the block with a linear regression fit to the performance accuracy of monkeys in the last 12 trials in the block. We computed the plateau performance accuracy, or *post-learning accuracy*, as the proportion of correct choices across all trials after the learning criterion was reached in a block (**Fig 3C and 3D**). In blocks in which the learning criterion was not reached, we computed the accuracy over the last 12 trials in the block. Analysis of eye movements used an adaptive velocity-based thresholding algorithm [8,9] to detect saccadic eye movements and fixations (*i*) onto objects during information sampling prior to choosing an object (*information sampling,* and **S7A-E Fig**), (*ii*) onto the chosen object prior to choosing it (*choice fixations*), and (*iii*) onto the token bar (*asset sampling*) (see e.g. **S1E, S1G, S1H** and **S2E, S2G and S2H Figs**). Fixation durations on objects indexing information sampling prior to choosing an object were shorter than 300 ms and well separated from *choice fixations* which had fixation durations of ≥700 ms (see the distribution of fixations in **S7 Fig**).

**Trial-level statistical analysis**.

We tested TUS effects on behavior at the trial level using linear mixed effects (LMEs) models [10] with 4 main factors: *cognitive load (Cog_Load_)* with three levels (1D, 2D, and 3D distractor feature dimensions, ratio scale with values 1,2 and 3), *trial in block (TIB)*, *previous trial outcome* (*Prev­_Outc)_*) which is the number of tokens gained or lost in the previous trial, motivational token condition, which we call the *motivational Gain/Loss context (*${MCtx}_{Gain/Loss}$*)* with two levels (1, for the loss condition, and 2 for the gain condition, nominal variable), TUS condition (${TUS}_{Cnd}$) with four levels (Sham-ACC, TUS-ACC, Sham-STR, TUS-STR), and *time relative to stim (T2Stim)* with 2 levels (before versus after stimulation). We used 3 other factors as random effects, a factor *target* *features* (*Feat*) with 4 levels (color, pattern, arm, and shape), weekday of the experiment (*Day*) with 4 levels (Tuesday, Wednesday, Thursday, and Friday), and the factor *monkeys* with 2 levels (W and I). We used these factors to predict 3 metrics (*Metric*): accuracy (*Accuracy*), reaction time (*RT*), information sampling (${Sample}_{Explr}$). The LME is formalized as in eq. 1.

$Metric={Cog}_{Load}+TIB+{Prev}_{Outc}+{MCtx}_{Gain/Loss}+ {TUS}_{Cnd}+T2Stim+\left( 1 | Day \right)+\left( 1 | Feat \right)+\left( 1 | Monkeys \right)+b+ \varepsilon$ (eq. 1).

**Analysis of behavioral adjustment to the recent token history (Gross Token Income)**.

To quantify how TUS affected choice accuracy depending on the motivational status of the subjects, we calculated the gross sum of the earned token over four trials which we call *gross token income* (GTI; **Figs 3F**, **S11E and S11F**). For each value of the GTI (spanning from – 3 to +6 tokens for gain-loss motivational context, and 0-9 tokens for gain-only motivational context conditions), we calculated the accuracy on the subsequent trial and normalized it by subtracting the mean and dividing by the standard deviation of the same GTI values during the baseline. We then asked whether the accuracy of choice for a given GTI was changed relative to baseline or other TUS conditions in any of the TUS conditions. We first used Wilcoxon signed-rank test to test for each TUS condition and each GTI, whether accuracy changes relative to baseline were different from zero. We then used FDR control of multiple comparison for dependent samples [11], with an alpha level of 0.05 to adjust p-values across different GTIs and the TUS conditions. In a second analysis, we applied randomization statistics to test whether, for a given GTI, the accuracy change was different compared to TUS conditions. We randomly permuted trial labels of the TUS conditions and selected 1000 subsamples with a sample size equal to the true TUS and GTI conditions. We then formed a randomized sampling distribution of the means and extracted the probability of randomly finding values more extreme than the true baseline-corrected accuracy value for each TUS and GTI condition, under the null hypothesis that for a given GTI none of the TUS conditions show accuracy changes relative to the baseline different than other TUS conditions. After finding the p-values for all GTI conditions, we corrected them for multiple comparisons using FDR control at an alpha level of 0.05. GTI values for a TUS condition were considered significant if it passed both tests (**Figs 3F**, **S11E and S11F**).

**Analysis of previous-trial-outcome effects on accuracy adjustment**.

We measured whether TUS modulated the accuracy on the following 1-5 trials after experiencing gain or loss outcomes (**Figs 3E** and **S11A-D**). Accuracy was baseline normalized for each TUS condition. Wilcoxon rank tests were applied to test the effects of the previous trial outcome after versus before TUS with FDR correction for dependent samples with an alpha level of 0.05. In a next step, we tested post-outcome accuracy changes in one TUS condition compared to other TUS conditions. Thus, for each outcome condition, we randomly permuted the TUS condition label of the trials, and randomly selected 1000 subsamples with a sample size equal to the size of the true outcome and the TUS condition (both in here and in the GTI analysis, only the TUS labels were shuffled and the rest of the labels for trials e.g., the order relative to TUS was still the same). We then used the 1000 subsamples and formed a randomized sampling distribution of the means and calculated the probability of finding a value more extreme than the true mean of changed trials accuracy following an outcome relative to the baseline, under the null hypothesis that the accuracy changes relative to the baseline after a given trial outcome is not different across different TUS conditions. After finding the p-values for each TUS condition and n^th^ (n=1-5) following trials, we used FDR correction for dependent samples with an alpha level of 0.05 to adjust p-values across different post outcome trials and the TUS conditions.

**Logistic fit to the performance accuracy**

As a supporting analysis to our measures on learning speed and performance accuracy we fit a logistic regression model to performance accuracy on each learning block using generalized linear mixed effects (GLMEs) models with a logit link function. We used the inflection point and asymptote and performed similar statistical analysis on them to compare these model driven values across different motivational contexts, cognitive load, and TUS conditions (**S8 Fig**). All main results stayed valid in the statistical analyzes.

**Pairwise comparisons of TUS effects.**

As a validation of the overall mixed effect modeling results, we report a Kruskal Wallis test for a main effect of TUS conditions on behavioral metrics and follow up with Wilcoxon tests for all pairwise comparisons between individual TUS conditions at each motivational context (gains-only and gains-loss). P-values were FDR corrected to control for multiple comparisons of dependent samples [11]. Complete statistical results are provided in **S1 Table**. We repeated the same procedure for pairwise comparisons of TUS effects at low cognitive load (1D condition) and high cognitive load (combining 2D and 3D conditions) (**S2 Table**).

**Randomization statistics for normalized behavioral metrics**.

We calculated the normalized value of a behavioral metric across blocks in each TUS condition, motivational contexts, and cognitive loads by subtracting their marginal means and dividing by the standard deviation. As shown in **Figs 2D** and **S5C** this procedure provides a table where each cell shows the mean of the marginally normalized metric value ± the standard error of normalized metric value (for each cell, the color shows from left to right the mean minus the standard error to the mean plus the standard error of that cell). We then performed randomization tests at two levels. First, at the level of the motivational contexts, we asked whether any TUS condition significantly differed from others by permuting the TUS condition labels and randomly selecting 1000 subsamples with a size equal to the size of the true TUS condition. This tested the null hypothesis that the marginally normalized metric values in a given TUS condition in a given motivational context do not differ from other TUS conditions in the same motivational context. We calculated the p-values and adjusted them post-hoc using FDR correction for dependent samples with an alpha level of 0.05. The white rectangles in **Figs 2D** and **S5C** show the FDR corrected, significant TUS. In a second analysis, we applied the same rationale and procedure but this time the randomization test was done on each motivational context and cognitive load condition separately to test the null hypothesis that TUS conditions had no effect on the behavioral metrics. Those TUS conditions that showed a significant difference from others in a given cognitive load and motivational context are marked with a white asterisk in **Figs 2D** and **S5C**.

**Session-Level analysis of TUS effects on behavioral metrics*.***

In addition to analyzing behavior in individual blocks and performing block-level statistics (see above), we also analyzed the data across sessions and provide these session-level results in **S1 Table** and **S10 Fig**. For each session, we calculated the mean of each behavioral metric for gain-only and gain-loss learning contexts separately before and after the TUS. We then used Wilcoxon tests for each TUS condition under the null hypothesis that the behavioral metric is not different before versus after TUS. To compare behavioral metrics across TUS conditions we first applied a Kruskal-Wallis test under the null hypotheses that the behavioral metrics did not differ between TUS conditions. Then we used pairwise Wilcoxon comparisons to compare each pair of TUS conditions. All p-values were corrected post-hoc using FDR for dependent samples with an alpha level of 0.05

**References**

1. Jenkinson M, Beckmann CF, Behrens TEJ, Woolrich MW, Smith SM. FSL. NeuroImage. 2012;62: 782–790. doi:10.1016/j.neuroimage.2011.09.015

2. Ungi T, Lasso A, Fichtinger G. Open-source platforms for navigated image-guided interventions. Medical Image Analysis. 2016;33: 181–186. doi:10.1016/j.media.2016.06.011

3. Chaplin V, Phipps MA, Jonathan SV, Grissom WA, Yang PF, Chen LM, et al. On the accuracy of optically tracked transducers for image-guided transcranial ultrasound. Int J CARS. 2019;14: 1317–1327. doi:10.1007/s11548-019-01988-0

4. Kim H, Chiu A, Park S, Yoo S-S. Image-guided navigation of single-element focused ultrasound transducer. International Journal of Imaging Systems and Technology. 2012;22: 177–184. doi:10.1002/ima.22020

5. Aubry J-F, Tanter M, Pernot M, Thomas J-L, Fink M. Experimental demonstration of noninvasive transskull adaptive focusing based on prior computed tomography scans. The Journal of the Acoustical Society of America. 2003;113: 84–93. doi:10.1121/1.1529663

6. Yang P-F, Phipps MA, Newton AT, Chaplin V, Gore JC, Caskey CF, et al. Neuromodulation of sensory networks in monkey brain by focused ultrasound with MRI guidance and detection. Sci Rep. 2018;8: 7993. doi:10.1038/s41598-018-26287-7

7. Munoz F, Meaney A, Gross A, Liu K, Pouliopoulos AN, Liu D, et al. Long term study of motivational and cognitive effects of low-intensity focused ultrasound neuromodulation in the dorsal striatum of nonhuman primates. Brain Stimulation. 2022;15: 360–372. doi:10.1016/j.brs.2022.01.014

8. Nyström M, Holmqvist K. An adaptive algorithm for fixation, saccade, and glissade detection in eyetracking data. Behavior Research Methods. 2010;42: 188–204. doi:10.3758/BRM.42.1.188

9. Voloh B, Watson MR, Konig S, Womelsdorf T. MAD saccade: statistically robust saccade threshold estimation via the median absolute deviation. JEMR. 2019;12. doi:10.16910/jemr.12.8.3

10. Pinheiro JC, Bates DM. Unconstrained parametrizations for variance-covariance matrices. Stat Comput. 1996;6: 289–296. doi:10.1007/BF00140873

11. Benjamini Y, Yekutieli D. False Discovery Rate–Adjusted Multiple Confidence Intervals for Selected Parameters. Journal of the American Statistical Association. 2005;100: 71–81. doi:10.1198/016214504000001907
