# Supplementary material for: Anterior cingulate cortex causally supports flexible learning under motivationally challenging and cognitively demanding conditions
Source: PLoS Biol. 2022 Sep 6;20(9):e3001785. doi: 10.1371/journal.pbio.3001785 (PMC9481162; doi:10.1371/journal.pbio.3001785)
Supplement: Attachment — Submitted filename: ReplyToReviewers_06.docx [file pbio.3001785.s015.docx]

**Summary of changes**

We are grateful for the constructive comments of the reviewers and address them point-by-point below. The revised manuscript includes extended new analyses, added results and figure panels, and multiple clarifications. The main changes include the following:

• We added results from simulations that quantify the ultrasound pressure in orbitofrontal cortex to rule out our effects are linked to OFC. The new **Suppl. Fig. S3F** shows that pressure in OFC was 27% or the pressure in ACC (158/574, see below), which suggests that the OFC unlikely contributes to the main effects.

• We added a new **Figure S7** to show that fixations on objects before the choice (indexing *information sampling*) and prolonged 700 ms fixations that were used to chose an object (indexing *choice fixations*) are well separated.

• We clarify that the main results are not dependent on the metric used to quantify the transition from learning to plateau performance. A newly added **Supplementary Figure 8** shows that fitting logistic regressions to accuracy provides results undistinguishable from quantifying learning as the trials-to-criterion performance.

• We added a new **Figure S11F** to show results of the GTI analysis for the gain-only context to enhance clarity of the analysis method.

• We added detailed information about **the actual peak negative pressure** for each of our target areas in the revised supplementary methods.

• We adjusted the revised in multiple ways, which included:

… adjusting the abstract and introduction to more fairly refer to a broader literature;

… avoiding statements of causality or necessity of the ACC for specific functions

to prevent overstating the implications of the results;

… using a consistent terminology to refer to object foveations as information

sampling;

… providing and explicit definition of how information sampling was

operationalized; and

… clarifying that each major result was visible separately in each animal as

explicitly shown in in monkey specific results in **Supplementary** **Figures S1-S11**;

Changes are visually highlighted with red font in the revised manuscript.

We wholeheartedly thank the reviewers for helping to improve the manuscript.

Sincerely,

Thilo Womelsdorf

Kianoush Banaie Boroujeni

Michelle Sigona

Louie Treuting

Thomas Manuel

Charles Caskey

**Point-by-point reply to reviewer comments.**

**Reviewer #1:**

_____________________________________________________________________

**Comment 1.**	This study wants to answer long standing questions within the reward learning literature about the roles of striatum and anterior cingulate cortex. To do this they use an elegant combination of a novel neurostimulation, Transcranial ultrasound stimulation (TUS), with a learning paradigm and thoughtful behavioural analyses in macaque monkeys. 
Specifically, the authors target two structures known to be involved in reward learning and decision making, anterior cingulate cortex (ACC) and striatum (STR) with their interfering neurostimulation and compare them to each other as well as two sham conditions.

 Somewhat surprisingly, they find behavioural impairments only after ACC stimulation, not STR. Moreover, only when the learning requires the macaques to consider potential losses as well as gains, does an impairment emerge.

When looking the data more carefully, they find that this is driven by only the data in which the animals need to ignore irrelevant dimensions, increasing the credit assignment problem. ACC stimulation, however does not only slow learning to criterion, but also reduces accuracy after criterion and leads to clustered errors.

Overall, the results are solid and very interesting to the field. I do not have any major suggestions regarding data analysis. However, some of the framing, particularly in the introduction and parts of the discussion is confusing. The authors spend a lot of time discussing different theories of ACC during decision making that only speak indirectly to their own results. Instead, there is a large ACC literature on reward learning and representations of different value dimensions (such as effort, loss and gains) as well as credit assignment that probably makes a more relevant framing.
I have tried to make my concerns and potential solutions as concrete as possible below.

*Reply: We very much appreciate the comments and suggestions of the reviewer. We believe addressing them in the revised manuscript helped to considerably enhance the clarity of results and interpretations. Thank you.*

_____________________________________________________________________

**Comment 2.** The authors very strongly emphasize two distinct ideas about ACC. One holds that ACC is involved in value guided decision, particularly when it comes to assessing alternatives, foraging, search and prospective planning. The other, claims ACC is exclusively concerned the signalling and resolution of conflict through control. However, while both theories have implications on what might happen during value guided learning, both are primarily concerned with decision making processes. It is not immediately clear how either theory would explain the learning deficits in a straightforward manner without some additions to the original theories. In fact, the current findings, if anything, are in direct contradiction with Conflict theories (in its many forms) because the primary function of ACC should have been linked to the load manipulation not the extension to the loss domain! Newer theories of conflict do include effort as a component but by no means suggest that losses are equivalent to effort and or that effort supersedes their postulated roles of ACC in general conflict resolution and signalling of difficult. In fact, the authors suggestion that their gain-loss condition is about effort seems rather post hoc to link their results to those theories, rather than being an established measure of effort. The other theory they talk about is at least neutral towards their findings and could easily be extended to consider ACC to not only have a role in decision making but also learning when situations are sufficiently ambiguous (high load) and there are mixed incentives, making it important to take into account undesirable hypotheticals. Additionally, theories emphasizing environmental value, are highly consistent with the observed clustering effects of low performance after ACC interferences, as ACC has been proposed to trigger behavioural change/switching/leaving of environments as a reaction to negative feedback or depleting patches.
Additionally, there are previous studies by Kennerley & Wallis on multiplexing (doi: 10.1038/nn.2961 ) and Hayden and Platt ( 10.1126/science.1168488 ) on hypothetical reward and ACC, that are nicely consistent with their findings (and the authors already cite). 
To cut a long story short, their results and study are better placed within the literature of reward learning, multiplexing of different value dimension and credit assignment, as their study clearly a reward learning one. Of course, mentioning other theories can be valuable but it gets confusing when the introduction is set up to put two theories against each other that are not directly about the behavioural measures presented in the results.

*Reply:* We thank the reviewer for this thoughtful comment. We agree with many aspects and re-wrote the abstract and introduction, and adjusted the discussion.

We rewrote the introduction to introduce explicitly why we designed a learning task that requires learning of feature values and varying the number of distracting features. We introduce three lines of evidence and conceptual frameworks that suggest specific mechanisms about how the ACC or striatum might use information about feature values to support learning, choices or information seeking. The introduction reduces the focus on the choice-value and effort control frameworks that we had in our initial submission and emphasizes the role of learning about feature values.

The introduction also makes more explicit why the experiment was designed to vary not only cognitive load, but also the motivational context.

In the discussion we start with a section that describes why our results are not necessarily contradicting conflict theories. We write:

*“… [I]ncreasing cognitive load in our task entailed increased perceptual interference from object features, but it did not entail a conflict of sensory-motor mappings that is the hallmark of conflict-inducing flanker, Stroop, or Simon tasks, used to document a role of the ACC to mediate the resolution of conflict (Ebitz et al., 2020; Botvinick et al., 2001; Mansouri et al., 2009). Our results therefore do not oppose studies using those paradigms and that gave rise to the view that neuronal signaling in ACC contributes to resolve sensori-motor conflicts (Heilbronner & Hayden, 2016).”*

Please see also the replies to other comments and the changes in the revised text (highlighted in red font).

_____________________________________________________________________

**Comment 3**.	Given ACC stimulation pretty much only leads to an impairment in the most challenging conditions (losses and gains combined with high cognitive load/multiple dimensions), one alternative explanation that should be mentioned is that it is simply a matter of impairment under the most challenging conditions, in which time to criterion is highest anyway. The authors, have two convincing pieces of evidence against this in the negative feedback specific effects and the higher errors even after criterion (although that could also be because it is just harder to remember with distractors and losses), but there should maybe be a brief mention of this more generic explanation of the results.

*Reply:* We agree and added two sections in the discussion to address this aspect. In a first section we write:

*“…The functional impairment in the gain-loss motivational context was most pronounced when subjects faced high cognitive load, i.e. in the condition that overall was most difficult. While this result pattern suggests that the overall difficulty may be the primary driver of the ACC-TUS effects, we outline below that the specific impairments after loss experiences (irrespective of cognitive load) and the overall reduced plateau performance with ACC-TUS in the gain-loss context indicate a predominant role of motivational processes over cognitive processes to drive the behavioral ACC-TUS effects. At a psychological level, the pronounced deficit in the loss context at high cognitive load is consistent with a neuroeconomic view of ACC function based on prospect theory, which suggest that losses induce a particularly strong internal demand for adjusting actions, which is intensified when the adjustment of the action itself gets more demanding by an increase in cognitive load [37,43,44].”*

*We continue discussing why we believe our results do not oppose conflict theories of ACC function by adding the following text to the revised manuscript:*

*“Our finding that experiencing losses was necessary to observe behavioral deficits entails that the presence of a cognitive ‘conflict’ due to increased number of features in the high cognitive load condition was not sufficient to alter performance. This result might seem at odds with a literature implicating the ACC to be particularly relevant to resolve conflict (Botvinick et al; 2001; Cole et al., 2010). However, increasing cognitive load in our task entailed increased perceptual interference from object features, but did not entail a conflict of sensory-motor mappings that is the hallmark of conflict-inducing flanker, Stroop, or Simon tasks, used to document a role of the ACC to mediate the resolution of conflict (Ebitz et al., 2020; Botvinick et al., 2001; Mansouri et al., 2009). Our results therefore do not oppose studies using those paradigms and that gave rise to the view that neuronal signaling in ACC contributes to resolve sensori-motor conflicts (Heilbronner & Hayden, 2016). Our results rather point to the importance of the ACC to contribute to overcoming situations in which motivational challenges require enhanced encoding of task-relevant variables.”*

_____________________________________________________________________

**Comment 4**.	Post error adjustment is a strange term for their effect after ACC stimulation. Isn't is more like a lack of adjustment? While non stimulated animals only make occasional or random errors, ACC stimulation leads to clusters of errors that a characterized by an inability to improve performance even after negative feedback. Maybe a better term would be "error clustering", "low performance periods" or "an inability to escape low average reward/negative feedback through behavioural change".

*Reply: We agree and thank the reviewer for pointing this out. This term appeared in the caption for figure 3. We changed instances of “post error adjustment” to more descriptively writing about “behavioral adjustment after gains and losses”. In the discussion we clarify that the effects are consistent with compromised credit assignment, i.e. the “…difficulty to assign negative outcomes to features of the chosen object…”.*

_____________________________________________________________________

**Comment 5.**	One slightly odd feature of the task is that both choices and "explorative sampling" are based on fixation. That makes it quite difficult to interpret the change of fixation duration as a change in explorative sampling as it could also be linked to changed decision making. It also gives another reason why criterion might take longer with added dimensions because they might inadvertently pick an option when they first look at it when there are many possible features to observe. In short, the authors should probably be careful with their strong interpretation of viewing duration given the specific features of the task and mention why that measures is a bit complicated.

*Reply:* We address this comment by adding new result figures in Figure S7 and by adding information in the revised text. We like to note that all fixational/explorative sampling is now termed “*information sampling*” throughout the revised manuscript following a suggestion of another reviewers.

In our task, subjects made a “response” by fixating an object for at least 300ms - when the fixation continued until 700ms since fixation onset the fixation was considered a choice of the fixated object (which could be correct or erroneous). When monkeys broke fixation between 300-700 ms the trial was aborted. The timing of this design was optimized during training to ensure that most (>90%) of ‘normal’ sampling fixation durations are shorter than 300ms which allowed separating choice-fixations from sampling fixations. In response to the reviewer comment we show the complete distributions of fixation durations in the new **Figure S7** to allow discerning this effect (for each monkey). We also show the bootstrap distributions to provide a statistical result that *sampling fixations* are shorter than 300ms and well separated from *choice fixations*.

We refer to the new **Figure S7** in the main text by writing …

*“…for detailed information about the distribution of fixational information sampling and its bootstrap sampling distributions for different motivational contexts, cognitive load, and TUS conditions, see* ***Figure S7****.”*

We also mention the separation of information sampling and choice fixations in the revised methods by writing…

*“Fixation durations on objects indexing information sampling prior to choosing an object were shorter than 300 ms and well separated from choice fixations which had fixation durations of ≥700 ms (see the distribution of fixations in* ***Figure S7****).”*

_____________________________________________________________________

**Comment 6**. Minor comments:

A)	The authors mention "Studies in humans have shown that stimuli associated with loss of monetary rewards or aversive outcomes (aversive images, odors, or electrical shocks) are less precisely memorized than stimulus features associated with positive outcomes (32-34)." There is also an interesting literature on planning and aversive pruning that might be relevant here. One example paper : doi:10.1371/journal.pcbi.1002410

*Reply:* This is an intriguing paper with multiple relevant aspects. Thank you for linking to it. We recognized that it is difficult to do due justice to the larger literature of punishments and losses for inhibiting behavior and curtailing the evaluation of options in sequential decision-making tasks (and our discussion is already >2500 words long). However, we added one key aspect of this literature to the discussion of the effects of losses on the specific encoding of precise object features. We now write in the revised manuscript:

*“Studies in humans have shown that stimuli associated with loss of monetary rewards or aversive outcomes (aversive images, odors, or electrical shocks) are less precisely memorized than stimulus features associated with positive outcomes (32–34). Such a reduced influence of loss-associated stimuli on future behavior may partly be due to loss experiences curtailing the engagement with those stimuli and reducing the evaluation of their role for future choices, as demonstrated, e.g., in a sequential decision-making task (Huys et al., 2012). One behavioral consequence of a reduced evaluation or memorization of aversive or loss-associated stimuli is an over-generalization of aversive outcomes to stimuli that only share some resemblance with the specific stimulus that caused the loss experience. Such over-generalization is evolutionary meaningful because it can support the faster recognition of similar stimuli as potentially threatening even if these stimuli are not a precise instance of a previously encountered, loss-inducing stimulus (Banaeie Boroujeni et al., 2022; Moscarello & Hartley, 2017).”*

_____________________________________________________________________

**Comment 7.**	B)	In Figure 2D the cells look multi-coloured, which is a bit confusing. Is there are way to change the colour scheme?

*Reply:* The individual cells are in fact multi-coloured to allow discerning the complete distributions of learning across blocks. We clarified in the legend that the colour gradient in each cell corresponds to values and variations across blocks. We write in the legend:

*“Each cell is color coded with the mean value ± SE with a low to high value gradient from left to right. Values across learning blocks in each cell are normalized by subtracting the mean and dividing by standard deviation of all baseline learning blocks across all TUS conditions for each load and motivational context.* *The white rectangle indicates that learning in that TUS condition (x-axis) is different from other TUS conditions. White asterisks indicate that a cognitive load condition in a learning context in a TUS condition is significantly different from other TUS conditions.”*

_____________________________________________________________________

**Comment 8.**	C)	Fig 3a unclear what the stat test is on. Is the only effect that comes out a test between aSTR stimulation and ACC stimulation?

*Reply:* In addition to the significant difference of TUS-ACC and TUS-STR we also found a significant difference of effects TUS-ACC to its pre-baseline period. To enhance clarity about he the meaning of the asterisks and horizontal line we adjusted the figure legend and now write:

*“Black asterisks show significant main effect of TUS conditions and a significant difference between the TUS and the baseline (pre-TUS) conditions (for the TUS condition underneath the asterisks). Horizontal black lines indicate significant pairwise difference between TUS conditions.”*

_____________________________________________________________________

**Comment 9.**	D)	I might be wrong here but it read as if the authors only do LME's for the plateau results. Is that correct and if so, why?

*Reply: LME’s were not only done for plateau results, but also for information sampling and learning (trials to criterion). We checked the methods section and ensured the LME’s are described for each of the dependent variables.*

####################################

**Reviewer #2:**

_____________________________________________________________________

**Comment 1.**	This study looks at the effects of focused ultrasound in the anterior cingulate cortex and anterior striatum in awake, behaving monkeys.  It has a number of strengths - 1. There are multiple targets and the behavioral effects depend on which target was sonicated, 2. Real and sham sonications were interleaved, 3. The dispersion of ultrasound pressure was modeled in 3D.  4. The behavioral task was rich enough to differentiate attention, motivation, and learning.  All facets of the study were well-executed.

*Reply:* Thank you for acknowledging these aspects of our study.

_____________________________________________________________________

**Comment 2.** There are 2 drawbacks - 1. The ultrasound pressure appears to spread into orbitofrontal cortex.  This could contribute the some of the effects.

*Reply:* In response to this point we now quantify with additional simulations the ultrasound pressure in OFC and compare it to ACC. We find the pressure in OFC was 27% or the pressure in ACC (158/574, see below), which suggests that the OFC unlikely accounts for the main effect. We modified and added a comparison to the **Supplementary Figure 3**. We write in the SI methods:

”*We ran a control analysis on ACC-TUS sessions, to assure that the effect is not attributed to modulation in orbitofrontal cortex (OFC) which appears aligned with the TUS orientation in* ***Figure 2.A****. From acoustic simulations we evaluated the pressure at the orbitofrontal cortex and compared it with the pressure at the anterior cingulate cortex. We centered a cube with an edge length of 4 mm around labeled targets at the ACC and OFC and calculated the spatial average within the volume. The mean spatial averaged pressure (mean ± standard deviation) for both NHPs was 574.1 ± 56.0 kPa at ACC (NHP W: 595.7 ± 46.3 kPa, NHP I: 555.3 ± 57.9 kPa) and 158.5 ± 56.6 kPa at OFC (NHP W: 137.3 ± 46.4 kPa, NHP I: 177.0 ± 59.1 kPa).“*

_____________________________________________________________________

**Comment 3.** The measurement and interpretation of some of the behavioral outputs, particularly, "information sampling," or "explorative sampling," is not well-described.  Both of these issues can be addressed by simply clarifying the text, without further experiments or analysis.

*Reply:* Thank you for pointing this out. We agree and more explicitly describe in the revised text how information sampling was measured and clarify the terminology by referring exclusively to information sampling (and not to explorative sampling).

We used two different metrics to index information sampling. One metric (which is shown in the figures) was the total duration of gaze fixations on objects prior to the gaze fixation onto the chosen object. As a second metric we used the number of fixations before the last fixation on the chosen objects. The two metrics were strongly correlated at r=0.914. We added this information more explicitly in the text and use the term *information sampling* throughout the revised text to avoid confusion:

“*We defined information sampling as the duration monkeys fixated objects prior to the last fixation in the trial that indexed choosing that object. This metric indexes how long information was processed about feature values of the objects prior to committing to a choice.*”

_____________________________________________________________________

**Comment 4.** Minor comments. It's helpful to include line numbers in the draft.

*Reply:* Line number are now added.

_____________________________________________________________________

**Comment 5.** The effects of sonication are described variously as "stimulation," or "disruption," and are compared to a lesion.  In other studies, ultrasound seems to enhance performance.  Could the authors discuss why they think ultrasound is disruptive?  Is this simply due to the current results, or are there other studies showing disruption?

*Reply:* The sonication protocol we applied was shown by Verhagen et al., 2019 to reduce the functional connectivity of the sonicated ACC region and interpreted as a disruption in that and follow-up papers from that group. We adopted their terminology. To address the reviewers concern we adjusted the text in the manuscript and use more descriptive terms such as “sonication” and “modulation” instead of “disruption” and discuss the findings in relation to the literature suggesting that the sonication protocol we used may link to a disruption at the systems level that may relate to a hyperpolarizing effects at the neural circuits level according to a recent in-vitro study we cite.

_____________________________________________________________________

**Comment 6.** The authors do not define "information sampling" nor do they specify the behavior that they interpret as information sampling.  It seems like it has something to do with fixation duration.  Is it just fixation duration or is there more to it?

*Reply:* We added the definition of information sampling in the revised text at an early place and more explicitly when we first describe the results that longer foveation durations on objects were evident in conditions with larger cognitive load. We now write

*”[…] longer foveation durations index more extensive information sampling of object features at higher cognitive load. We defined information sampling as the duration monkeys fixated objects prior to the last fixation in the trial that indexed choosing that object. This metric indexes how long information was processed about feature values of the objects prior to committing to a choice.“*

*And in the discussion*

*“Longer fixation durations are typically considered to reflect longer sampling of information from the foveated object. This has been inferred from the longer fixational sampling of objects associated with higher outcome uncertainty [49], or that explicitly carry information that reduces uncertainty about outcomes [50,51]. We, therefore, consider fixational sampling of visual objects to index information sampling. Consistent with this view, we found that ACC-TUS prolonged information sampling at high cognitive load when objects varied in more features from trial to trial and hence carried higher uncertainty about which feature was linked to reward (****Figure 2D****,****E, Figure S7****). …”*

_____________________________________________________________________

**Comment 7.** It seems like learning rate was estimated by "trials to criterion" and, sometimes, linear extrapolation.  A better approach is to fit a logistic regression with rate and asymptote parameters.

*Reply:* We did estimate the trial at which learning transitioned to plateau performance with a fitting procedure in previous studies and did not find it in our task paradigms to be superior to the model-free trials-to-criterion metric. However, to test this explicitly we followed the suggestion and fitted a logistic regression model using GLM on blocks of learning. We compared the inflection points and asymptotes from the model fit. We repeated our main statistical analyses on these new measures and all our previous observation held true. We however did not change the previous measures as all figures were plotted based on the trials-to-criterion metric.

We added a paragraph in the revised text explaining the model fit and also added a new supplementary figure showing the model fit to the performance accuracy data across different motivational context, cognitive load, and TUS conditions (**Figure S8**). We write:

*”We confirmed these results using a second metric to estimate learning and plateau performance by fitting logistic GLMs to performance accuracy in each block (all previously significant results for trial-to-criterion and plateau accuracy remained significant when instead comparing inflection point and asymptote,* ***Figure S8****).*”

_____________________________________________________________________

**Comment 8.** The idea that ultrasound effects are seen mainly in low-motivation conditions is consistent with results recently published by Munoz et al. in Brain Stimulation.

*Reply:* Thank you for linking to this paper. We believe this study will become relevant for future studies when we extent the sonication focus to different subregions of the basal ganglia. We refrain from adding a discussion section on this study as the stimulated brain areas, stimulation modality, and the focus of the antisaccade paradigm are different and largely indirectly related to our study. We use and reference the linked paper to help understand the acoustic stimulation values described in the methods section.

_____________________________________________________________________

**Comment 9.** It isn't clear how many sonications and shams were done in each target region per monkey.  Based on the supplement, it appears to be 6 sonications and 6 shams per target per monkey.  Please confirm if this is correct.

*Reply:* Thank you for pointing us to this. We sonicated 12 times per condition (not 6 times) and clarify this early in the results section of the revised text by writing:

“*We performed 12 experimental sessions for each TUS or sham condition in each of the two brain areas ACC or STR (a total of 48 sessions) with each of the two monkeys.*”

_____________________________________________________________________

**Comment 10.** Please report the absolute de-rated peak negative pressure.

*Reply:* We thank the reviewer for suggesting this and added this information to the SI Method and two supporting references:

”*From acoustic simulations we determined the spatial averaged pressure was 581.5 ± 95.8 kPa (NHP W: 623.38 ± 124.93 kPa, NHP I: 544.79 ± 31.16 kPa) at the striatum and 574.1 ± 56.0 kPa at the ACC (NHP W: 595.7 ± 46.3 kPa, NHP I: 555.3 ± 57.9 kPa). We used these pressures to estimate a mean attenuation of 51.9% for both NHPs (NHP W Striatum: 48.1%, NHP W ACC: 50.4%, NHP I Striatum: 54.6%, NHP I ACC: 53.7%). These values agree with prior estimations of macaque skull attenuation based on water bath measurements previously reported by our group and others (Yang, Pai-Feng, et al 2018, Munoz, F., et al, 2022).”*

####################################

**Reviewer #3:**

_____________________________________________________________________

**Comment** The anterior cingulate cortex and the striatum are both implicated in learning and sequential decision-making, but causal evidence is sparse, especially in the primate. The study thus fills an important gap in understanding the causal links between activity in these structures and the process of learning feature-reward rules. The study applied transcranial ultrasound stimulation to these two structures and evaluated behavioral indices of learning in a task which manipulates motivational context (gain-only, gain-loss) and attentional and/or cognitive load (low, medium, high). After stimulation to anterior cingulate cortex in the gain-loss context, learning was slower, asymptotic accuracy was lower, and gaze duration before the choice was longer. The context-specificity of these effects are likely due to the fact that ACC stimulation selectively disrupted performance following losses, consistent with a large literature linking this structure to post-error compensatory processes. The fact that slower learning was driven by the higher cognitive load condition also resonates with older literature linking this region to adaptation in the face of conflict and cognitive demands. By providing causal evidence for long standing theories that link ACC to post-error and performance monitoring functions, this study could potentially be a valuable contribution to the literature. However, it would benefit from discussing this literature, including an additional control analysis, improving the precision of the text, a thorough copy edit, and an acknowledgement of a few of the limitations of the results and methods.

*Reply:* We thank the reviewer for the concise and positive summary – and for pointing us to various aspect that deserved enhancements and clarifications.

_____________________________________________________________________

**Comment 1.** It is difficult to understand the results obtained with the use of linear mixed effect (LME) models. A few examples:

"Learning is slowed down (trials-to-criterion increased) after TUS in ACC in the gain-loss context (left, LME's, p=0.007) but not in the gain-only contexts (right, n.s.)." (Figure 2B)

"TUS in ACC but not sham-TUS in ACC or TUS or sham-TUS in the striatum (Figure 2A) changed this behavioral pattern. ACC-TUS selectively slowed learning in the gain-loss contexts compared to the gain-only contexts (LME's, t=2.67, p=0.007)" (p. 5)

Is this the interaction between the motivational context and the TUS condition? Please specify what term is being reported here and for all linear mixed effects models. Please also add the reports in other places, where it seemed that a linear mixed effect model was being used, but only the post hoc comparisons were reported (i.e. p.6, lines 8-11).

*Reply:* Thank you for pointing us to this oversight on our end. We adjusted the revised text and make explicit what type of main effect or interaction is meant for each reported LME result. We now write, e.g.

*“TUS condition showed a significant interaction with motivational context with ACC-TUS selectively slowing learning in the gain-loss contexts compared to the gain-only contexts (LME interaction of TUS condition and Motivation Context, t=2.67, p=0.007) (****Figure 2B****;* ***Figure S4A****,****B, Table S1****).”*

We also simplified the terminology of the TUS conditions throughout the revised manuscript to ACC-TUS , ACC-Sham, STR-TUS, and STR-Sham.

_____________________________________________________________________

**Comment 2.** In causal NHP papers, it is standard to report effects individually for each animal, particularly when there are only 2 animals used. It seems that the effects reported here may not have been individually significant (though this information was not provided, at least in the main text). This doesn't necessarily mean that the results are not interpretable, but it is a limitation that should be commented on in the discussion.

*Reply:* We think this was a misunderstanding that we clarify in the revised text*.* We provided the results for each monkey separately in the extended supplementary figures including the effects of cognitive load and motivational context (Fig. S1+ S2), the sonication parameters (Fig. S3), the learning and information sampling effects of TUS conditions and cognitive load and motivational contexts (Fig. S4, S5, and S6), time course analysis (Fig. S9) and the Session level analysis that we added as a control (Fig. S10). The results are overall consistent across animals with no specific discrepancy.

We also added a newly added results about the distribution of information sampling for each monkey separately (Fig. S7) and a control analysis of learning requested by reviewer 2 (Fig. S8).

We show these monkey specific findings in the supplementary material and not the main text as they would more than double the figure panels needed to convey the results which is not necessary as they support the main findings without an apparent qualitative discrepancy that would deserve a separate discussion in the main text. We added a sentence to the discussion of the revised manuscript to make this aspect more explicit by writing

*“… that the main TUS-ACC effects were qualitatively consistent across monkeys (for monkey specific results, see* ***Figures S1-S10****; there were no significant random effects of the factor monkey in the LME models).*

_____________________________________________________________________

**Comment 3.** Simulation was the only evidence provided for the efficacy of ultrasound stimulation and there was no experimental verification that the procedure changed the neural activity within the targeted structures. This is a major concern for ultrasonic stimulation since we still know little about its impact on neuronal activity. In the absence of some kind of experimental verification that the effects of stimulation were directional and localized in ACC (vs affecting fibers of passage or connected regions, for example), the authors should eliminate all inferences about the specificity and directionality of the effects (i.e. "ACC is necessary", "disrupting ACC", etc.). These claims occurred throughout the manuscript. The limitations in these causal interpretations also need to be made clear in the discussion.

*Reply:* We agree and adjusted the text at multiple places. Instead of referring to TUS as “disrupting ACC”, we now refer to the TUS protocol to “…*modulating neural activity…*” or similar.

In addition, before describing the results, we make explicit that the sonication protocol used has previously been shown to “… reduce functional connectivity of the sonicated area in macaques (18), and in in-vitro preparations to modulate neuronal excitability to external inputs (21).”

We also …

- avoid writing ‘*TUS directed to ACC’* and now say ‘*TUS of ACC*’;
- avoid writing ‘*ACC is necessary*’ and say instead ‘*ACC contributes*’.

In response to the comment of reviewer we also added text in the discussion to highlight the limitations of knowledge of TUS effects on neural circuits by writing:

*“Prior to discussing the implications of these findings it should be noted that the sonication effects on neuronal activity in-vivo are not well investigated and may involve changes in the excitability of neural tissue within the hot spot of the sonicated area [41], or effects on the fibers of passage through the sonication areas. Our discussion assumes a putative disruptive effect of TUS on neural activity within ACC. This assumption is based on a prior study that used the same TUS protocol and reported reduced functional connectivity of the area sonicated [38]. There is also the caveat that the sonication effects could indirectly follow altered activity in other areas through altering connectivity with those areas. “*

We also extended the discussion section entitled “*Versatility of the focused-ultrasound protocol for transcranial neuromodulation*” and point out that the circuit mechanisms of the FUS effects are not well understood by writing:

“*Despite these suggestions it should be made explicit that there is so far a scarcity of insights about the specific effects of the TUS protocol on neural circuits. Future studies will therefore need to investigate the neural mechanism underlying the immediate and longer-lasting TUS effects on ACC neural circuits.*”

_____________________________________________________________________

**Comment 4.** The loss-specificity of the results is an important part of the contribution of this paper, but the method used to show that ACC-TUS effects were specific to the losses appears to combine data in an odd way--combining the gain data between both contexts (one in which there was an effect and one in which there was not), rather than comparing gain and loss within the same gain-loss context. If I'm understanding this correctly, it would be helpful to perform an analysis analogous to Fig 3F and S9E for the gain-loss context only.

*Reply:* Thanks for pointing this out and suggesting we had not described this sufficiently well. Contrary to what the reviewer thought, the analysis was done already in a context-specific way (it was done only for trials in gain-loss context). We clarify this in the figure caption as well as in the revised results section, writing:

“ACC-TUS reduced performance accuracy on trials in the gain-loss context specifically when subjects had lost 2 or 3 tokens in the preceding four trials, but not when their net token gain in the past four trials was ≥0 tokens, and not for trials in gain-only context (random permutation test, p<0.05, **Figure 3F, Figure S11F**).”

To enhance clarity, we also added similar plots and analysis for GTI in the Gain-only conditions to the figure in a new panel **Figure S11F**.

_____________________________________________________________________

**Comment 5.** The loss-specificity and interaction with cognitive demands of the ACC-TUS stimulation resonate with a body of literature that links ACC to performance/error monitoring and compensatory functions. However, there was no discussion of these classic ideas and theories. It would be very helpful to situate these results in a proper historical context, rather than focusing the literature review on recent papers with tenuous relevance to these results.

*Reply:* We address this concern by rewriting the introduction to make more explicit the rationale for the study and for adding a section to the revised discussion that explicitly considers how our results link to performance/conflict monitoring theories of the anterior cingulate cortex, and by clarifying why we are structuring the discussion the way we do.

We added a new section at the beginning of the discussion summarizing more explicitly the main pattern of results (i.e. the effects of information sampling and the response to loss experiences) and how performance monitoring models might relate to it. We added:

*“Our finding that experiencing losses was necessary to observe behavioral deficits from ACC-TUS shows that the presence of a cognitive conflict due to an increased number of features in the high cognitive load condition was not sufficient to alter performance. This result might seem at odds with literature implicating the ACC to be particularly relevant to resolving conflict [46,47]. However, increasing cognitive load in our task entailed increased perceptual interference from object features, it did not entail a conflict of sensory-motor mappings that is the hallmark of conflict-inducing flanker, Stroop, or Simon tasks, used to document a role of the ACC to mediate the resolution of conflict [26,46,48]. Therefore, our results do not oppose studies using those paradigms, which gave rise to the view that neuronal signaling in ACC contributes to resolving sensori-motor conflicts [2]. Our results instead point to the importance of the ACC in contributing to overcoming situations in which motivational challenges require enhanced encoding of task-relevant variables.”*

We think the next four sections of the discussion are well justified to help constraining the possible implications of our findings using relevant, recent literature. In particular, we discuss in separate sections:

• ACC modulates the efficiency of information sampling

• ACC mediates feature-specific credit assignment for aversive outcomes

• A role of the ACC to determine learning rates for aversive outcomes

• Versatility of the focused-ultrasound protocol for transcranial neuromodulation

The length of the discussion is ~2500 words and may already be at the longer end for this journal.

_____________________________________________________________________

**Comment 6.** Minor issues: - When discussing the contextual change introduced by gain-only and gain-loss conditions, the terms "reward structure", "motivational/affective demands" and "payoff" are used interchangeably. This made it harder than necessary to understand the actual manipulations in the task. The interchangeability of these various descriptors also meant that the results occasionally veered off into speculative directions where the logic was tough to follow.

*Reply:* Thank you for pointing us to these inconsistencies. We adjusted the text at the respective places to be more consistent.

_____________________________________________________________________

**Comment 7.** - The use of the term "exploration" in this paper was confusing. In the context of sequential decision-making tasks like this one, exploration has a technical meaning: a kind of non-exploitative decision focused on learning about the options. However, manuscript sometimes uses "explorative sampling" to refer to gaze duration. (Please note that the manuscript also sometimes used the term "fixational sampling" directly to talk about "explorative sampling" [e.g. p. 9].) It would be more clear to replace "explorative sampling" with a less loaded term, like "information sampling", as is already done in some parts of the text. This is particularly important because the text does refer to the explore/exploit dilemma in the traditional way elsewhere (i.e. p. 6).

*Reply:* We agree and are grateful the reviewer points this out. We adjusted the revised text to use “*information sampling*” instead.

_____________________________________________________________________

**Comment 8.** - The term "attentional load" seems misplaced as the role of attention in this task is not clear. Increasing the number of stimulus features could increase load at a variety of cognitive levels, not just at the level of attention, and none of the analyses presented here suggest that this manipulation alters attention. Maybe "cognitive load" would be better?

*Reply:* In response to this comment we changed the text, figure, and supplementary material to refer to “*cognitive load*” throughout the revised paper.

_____________________________________________________________________

**Comment 9.** - In the text, TUS conditions are called ACC-TUS, ACC-sham, aSTR-TUS, aSTR-sham, but in the figures they are called H-ACC, S-ACC, H-aSTR, S-aSTR. This second set of abbreviations is only explained in the supplement (Sham-ACC, High-ACC, Sham-STR, High-STR). 

*Reply:* Thank you for pointing us to this issue and apologies for the confusion. We changed all labels in the figures and made all condition names consistent in the revised manuscript.

_____________________________________________________________________

**Comment 9.** - Notation was inconsistent for a variety of other terms throughout the manuscript and supplement (e.g. "LMEs" and "LME's", "t" and "t-stat", "gain/loss condition" and "gain-loss condition", "STR" and "aSTR").

*Reply:* Thank you. We carefully adjusted the text to use consistent terms.

_____________________________________________________________________

**Comment 10.** - Definitions for some acronyms were missing entirely from the main text and found only in the supplement.

*Reply:* Thank you. We now define the definitions of acronyms in the main body of the revised text.

_____________________________________________________________________

**Comment 11.** - Mean values of two major dependent variables are missing in the text (plateau accuracy and information sampling), though mean values of learning time are reported.

*Reply:* We added the mean values and write

“*… mean plateau accuracy was reduced to 84.5%±1.4 (monkey W/I: 85%±1.4 / 84%±1.4) relative to the pre-TUS baseline (plateau accuracy: 88±1.8; monkey W/I: 88±2 / 88±1.6 …*”

and

“*… ACC-TUS information sampling (ms): 234±6 ms monkey W/I: 230±6 / 237±7 ms, pre-TUS information sampling (ms): 209±11 ms monkey W/I: 197±16 / 221±11 ms; …*”

_____________________________________________________________________

**Comment 12.** - In the Supplement: "Where appropriate we used a non-parametric approach by fitting generalized linear mixed effects models (GLME's)" [...] "we used a link Identity function, and a Poisson distribution for the response variable." A model that makes an assumption about the distributions is not non-parametric. Also, a log link would be more common for a Poisson distribution because the distribution is undefined for negative parameters. The use of a non-standard link function should always be justified.

*Reply:* Thank you for mentioning this. The reviewer’s point is valid that it can yield negative values. It was intended to be a non-normal distribution and we mistakenly forgot to remove it as we no longer used GLM (but the newly added logistic regression model fit) in this study. We removed this line, and only explained the newly added analysis in response to a request of reviewer 2.

_____________________________________________________________________

**Comment 13.** - p. 5 last paragraph "(LME's, t=2.67, p=0.007))" - extra parenthesis
- p. 6 first paragraph - reference to Figure 3C,D. Is this actually referencing Figure 2C,D?
- The use of the acronyms GLME and LME was a bit unusual. I couldn't find their definitions in the main text, but in the supplement, they were defined as "linear mixed effect models (LME)", "generalized linear mixed effects models (GLME's)". Typically these would be abbreviated as "Linear Mixed Effects (LME) models" or else could be extended to "LMEMs" or "GLMEMs" for brevity.

- *Reply:* We corrected this in the revised text.

_____________________________________________________________________

**Comment 14.** Figure 1: the small table presenting motivational contexts and axes presenting task dimensions are not referenced by the caption and the panel is unlabeled.

*Reply:* We clarified the axes in the caption:

*“The axes show the three orthogonal independent variables of the task design (cognitive load, motivational context, and TUS conditions).*”

We added a label for the table and referenced it now.

_____________________________________________________________________

**Comment 15.** - Figure 2B: it is not clear what is meant by the horizontal lines and the star.

*Reply:* We clarified the meaning in the figure caption:

“*Black Crosses (×) and asterisks mark significant interactions of cognitive load and TUS conditions. Black asterisks indicate significant main effects of TUS conditions and a significant difference between the TUS and the baseline (pre-TUS) conditions (for the TUS condition underneath the asterisks). Horizontal black lines indicate significant pairwise difference between TUS conditions.”*

_____________________________________________________________________

**Comment 16.** - Figure 2C: what is represented by * and X ?

*Reply:* Please see previous comment. They represent significant interactions.

_____________________________________________________________________

**Comment 17.** - Figure 2D: what does "marginally normalized trials-to-criterion" mean? (labeled "Diff. Norm. Trial-to-criterion" in the figure)

*Reply:* It refers to the difference of values in this test from the baseline which is normalized to the marginal mean and standard deviation. We outline in the method that we normalized values in each of the cells by subtracting the mean of values across all TUS conditions during the baseline period within the same motivational context and each cognitive load (as the cell), and dividing by its standard deviation. We added a clarification in the figure legend and write:

”*Values across learning blocks in each cell are normalized by subtracting the mean and dividing by standard deviation of all baseline learning blocks across all TUS conditions for each load and motivational context.”*

_____________________________________________________________________

**Comment 18.** - Figure 3: it might help to maintain a common convention with the Figure 2 (i.e. gain-loss and gain-only results could be presented under the same letter). There are also more ambiguous symbols here that are not defined in the caption.

*Reply:* Thank you, we clarified all symbols now.

_____________________________________________________________________

**Comment 19.** - Figure S4A - typo in the title ("gain-loss")

*Reply:* corrected.

_____________________________________________________________________

**Comment 20.** - Figure S5A - significance marked for monkey W but not in both monkeys together? Is this different from Figure 2C presenting the same result?

*Reply:* Thank you for finding this issue. It was actually the same result, only the significant marks were overlooked. They are added now.

_____________________________________________________________________

**Comment 21.** - Figure S6E - missing significance star in both monkeys? Is this different from Figure 3C presenting the same result?

*Reply:* Similar to the previous comment. Corrected now.
